# Left ventricular systolic dysfunction predicts incremental utility of delayed enhancement CMR vs. echocardiography for diagnosis of LV thrombus

**DOI:** 10.1186/1532-429X-11-S1-P140

**Published:** 2009-01-28

**Authors:** Kirsten O Healy, Jason Chinitz, Raymond J Kim, Aqsa Shakoor, Michael I Ross, Matthew D Cham, James K Min, Michele Parker, Mary J Roman, Richard B Devereux, Jonathan W Weinsaft

**Affiliations:** 1 Weill Medical College of Cornell University, New York, NY USA; 2 Duke Cardiovascular Magnetic Resonance Center, Durham, NC USA

**Keywords:** Systolic Heart Failure, Lower Ejection Fraction, Left Ventricular Thrombus, Remodel Index, Initial Screening Test

## Background

Delayed enhancement cardiac magnetic resonance (DE-CMR) imaging is well-validated as a highly accurate tool for left ventricular thrombus (LVT). In prior research studies, DE-CMR has been shown to provide improved diagnostic performance vs. echo for detection of LVT. However, in clinical practice, echo is widely used as an initial screening test for LVT. Identification of echo imaging variables that predict added utility of DE-CMR is important for optimization of clinical imaging strategies for LVT.

## Methods

We studied markers that predicted improved LVT assessment by DE-CMR among patients with systolic heart failure or myocardial infarction enrolled in a multimodality imaging study. CMR and echo were performed within a seven day interval (Δ0.8 ± .2 days) and interpreted blinded to results of the other modality. Echo included both non-contrast echo (NC-echo) and contrast echo (C-echo) imaging in all patients. CMR (1.5 T) included cine-CMR (SSFP) and DE-CMR (inversion recovery; standard TI = 250–350 msec/long TI = 600 msec). DE-CMR was the reference standard for LVT. Left ventricular (LV) function and geometry were quantified by cine-CMR (planimetry) and echo (linear) methods. Infarct size was measured on DE-CMR. Echoes were graded for image quality on a uniform scale to account for endocardial definition, artifacts, and number of segments imaged.

## Results

130 patients were studied (62 ± 13 yo, 98% CAD, NYHA class 2 ± .8). LVT prevalence was higher by DE-CMR than C-echo (21% vs. 14% p < 0.05). Patients with LVT had larger infarcts (OR 1.5 per 10% LV p = 0.01), lower ejection fraction (OR 1.9 per 10% EF p = 0.01) and more aneurysms (OR 3.3 p < 0.05). Echo performance did not vary by clinical parameters or image quality, with similar reader-assigned quality scores between NC-echoes that did or did not detect LVT (7.0 ± 1.9 vs. 6.8 ± 1.7, p = .8). Ejection fraction was lower among patients that derived improved LVT assessment by C-echo compared to those in whom NC-echo alone accurately assessed LVT (32.1 ± 10.7% vs. 39.9.0 ± 13.4%, p < 0.05). Similarly, improved LVT assessment by DE-CMR was associated with lower ejection fraction and greater cavity dilation as measured by either echo or cine-CMR (Table 1). In multivariate analysis, severity of EF impairment (OR 1.8, p < 0.05) was an independent marker for added utility of DE-CMR after controlling for LV volume.

**Table 1:** Echo performance in relation to LV geometry

		NC-Echo Concordance with DE-CMR	NC-Echo Discordance with DE-CMR	P
**Echo Parameters**	Ejection fraction (%)	41.2 ± 13.6	32.6 ± 9.8	0.003
	End-systolic diameter (cm)	4.7 ± 0.9	5.2 ± 0.9	0.03
	End-diastolic diameter (cm)	5.9 ± 0.7	6.1 ± 0.8	.24
	Aneurysmal Dilation	13.5%	15.4%	.80
**Cine-CMR Parameters**	Ejection fraction (%)	40.8 ± 13.8	31.2 ± 12.0	.002
	End-systolic volume (ml)	113.0 ± 68.6	152.0 ± 81.9	0.02
	End-diastolic volume (ml)	180.9 ± 68.9	211.1 ± 82.9	0.06
	Aneurysmal Dilation	10.6%	23.1%	0.09

## Conclusion

LV remodeling indices are useful for guiding imaging strategies for LVT. Patients in whom DE-CMR provides incremental utility for LVT detection vs. NC-echo have more advanced LV dysfunction than those in whom LVT is accurately assessed by NC-echo alone.

